# Hypolipidemic effect of chromium-modified enzymatic product of sulfated rhamnose polysaccharide from *Enteromorpha prolifera* in type 2 diabetic mice

**DOI:** 10.1007/s42995-022-00127-0

**Published:** 2022-04-05

**Authors:** Xinyu Wang, Han Ye, Jiefen Cui, Yongzhou Chi, Ruizhi Liu, Peng Wang

**Affiliations:** 1 College of Food Science and Engineering, Ocean University of China, Qingdao, 266003 China; 2 State Environmental Protection Key Laboratory of Estuarine and Coastal Environment, Beijing, 100012 China

**Keywords:** Sulfated rhamnose polysaccharides, *Enteromorpha prolifera*, Chromium, Hypolipidemic, Type 2 diabetic mice

## Abstract

Sulfated rhamnose polysaccharide (SRP) derived from *Enteromorpha prolifera* is a metal-ion chelating agent that could potentially be used to treat diabetes. The aim of our study was to determine the effect of a variant of SRP on DIABETES. First, we synthesized and characterized SRPE-3 chromium(III) [SRPE-3-Cr(III)] complex using an enzymatic method. The maximum chelation rate was 18.2% under optimal chelating conditions of pH 6.0, time 4 h, and temperature 60 °C. Fourier transform infrared spectroscopy results showed important sites for Cr(III)-binding were O–H and C=O groups. We then studied the hypolipidemic effects of SRPE-3-Cr(III) on type 2 diabetes mellitus (T2DM) induced by a high-fat, high-sucrose diet (HFSD). Decreased blood glucose content, body fat ratio, serum TG, TC, LDL-C, and increased serum HDL-C were observed after treatment with SRPE-3-Cr(III). In addition, SRPE-3-Cr(III) significantly reduced leptin, resistin, and TNF-α levels, and increased adiponectin contents relative to T2DM. Histopathology results also showed that SRPE-3-Cr(III) could alleviate the HFSD-lesioned tissues. SRPE-3-Cr(III) also improved lipid metabolism via a reduction in aspartate aminotransferase, alanine aminotransferase, fatty acid synthase, and acetyl-CoA carboxylase activities in the liver. SRPE-3-Cr(III) at low doses exhibited better lipid-lowering activities, hence, could be considered to be a novel compound to treat hyperlipidemia and also act as an anti-diabetic agent.

## Introduction

Type 2 diabetes mellitus (T2DM) is an increasing global health problem (Chen et al. 2019). T2DM is due to reduced insulin secretion from pancreatic β cells or insensitivity to the peripheral actions of insulin (Saravanakumar et al. 2021). Such insulin insensitivity is often exacerbated by excessive lipid deposition (Franks and Mccarthy 2016). Dyslipidemia is a common complication of hyperlipidemia, leading to the increase in free fatty acids, which can aggravate insulin resistance (Bagri et al. 2009; Zhao et al. 2020). Thus, most patients with diabetes need a combination of anti-diabetic medications to control their dyslipidemia. Available anti-diabetic drugs lower blood glucose levels through different mechanisms. However, use of these drugs is limited by their tolerance and safety. In addition, anti-diabetic drugs alone are not sufficient to control dyslipidemia (Suksomboon et al. 2014).

Chromium plays an important role in carbohydrate and lipid metabolism (Feng et al. 2018; Guo et al. 2020). Chromium deficiency is reported to be associated with insulin resistance and diabetes (Zhang et al. 2010). Various organic forms of Cr exist, with different forms having different bioavailability (Mowat 1994). Ligands can enhance the bioactivity of chromium complexes possibly better controlling diabetes. Wang et al. (2017) found that Cr supplemental as *Inonotus obliquus* polysaccharides–chromium(III) complex could decrease lipid peroxidation, improve impaired glucose tolerance, and reduce tissue damage in T2DM mice.

Sulfated rhamnose polysaccharide (SRP) derived from *Enteromorpha prolifera* is a group of heteropolysaccharides possessing significant bioactivities, such as antitumor, antiviral, and anticoagulant properties (Cui et al. 2018). Because of the negative charge of the sulfated polysaccharide and its ion-exchange capacity, the polysaccharide can act as a ligand for metal binding to exert synergistic biological activities (Chi et al. 2018; Wang et al. 2019). For example, Cui et al. (2019) prepared a rhamnan-type sulfated polysaccharide derivative, demonstrating that it could ameliorate the diabetic symptoms through the PI3K/PKB/GSK-3β signaling pathway. Ye et al. (2019) also reported that the sulfated rhamnose polysaccharides’ chromium(III) complex exhibited great hypoglycemic capacities in high-fat, high-sucrose diet (HFSD)-induced T2DM mice. However, to date, the effect of conditions including pH, temperature, and time on the chelation of SRP and hypolipidemic effect of SRP chromium(III) remain poorly understood.

In this study, the enzymatic product of SRP was first prepared then used to synthesize a chromium(III) complex. Next, its hypolipidemic activity in T2DM mice was evaluated. The synergistic effect between chromium, with its variety of biological activities, and the polysaccharide, with its unique structure and bioactivities, could be used to control blood lipid levels, hence be a promising alternative to treat T2DM.

## Results

### Purification of enzymatic product of SRP and characterization of SRPE-1, SRPE-2, and SRPE-3

**Fig. 1:**
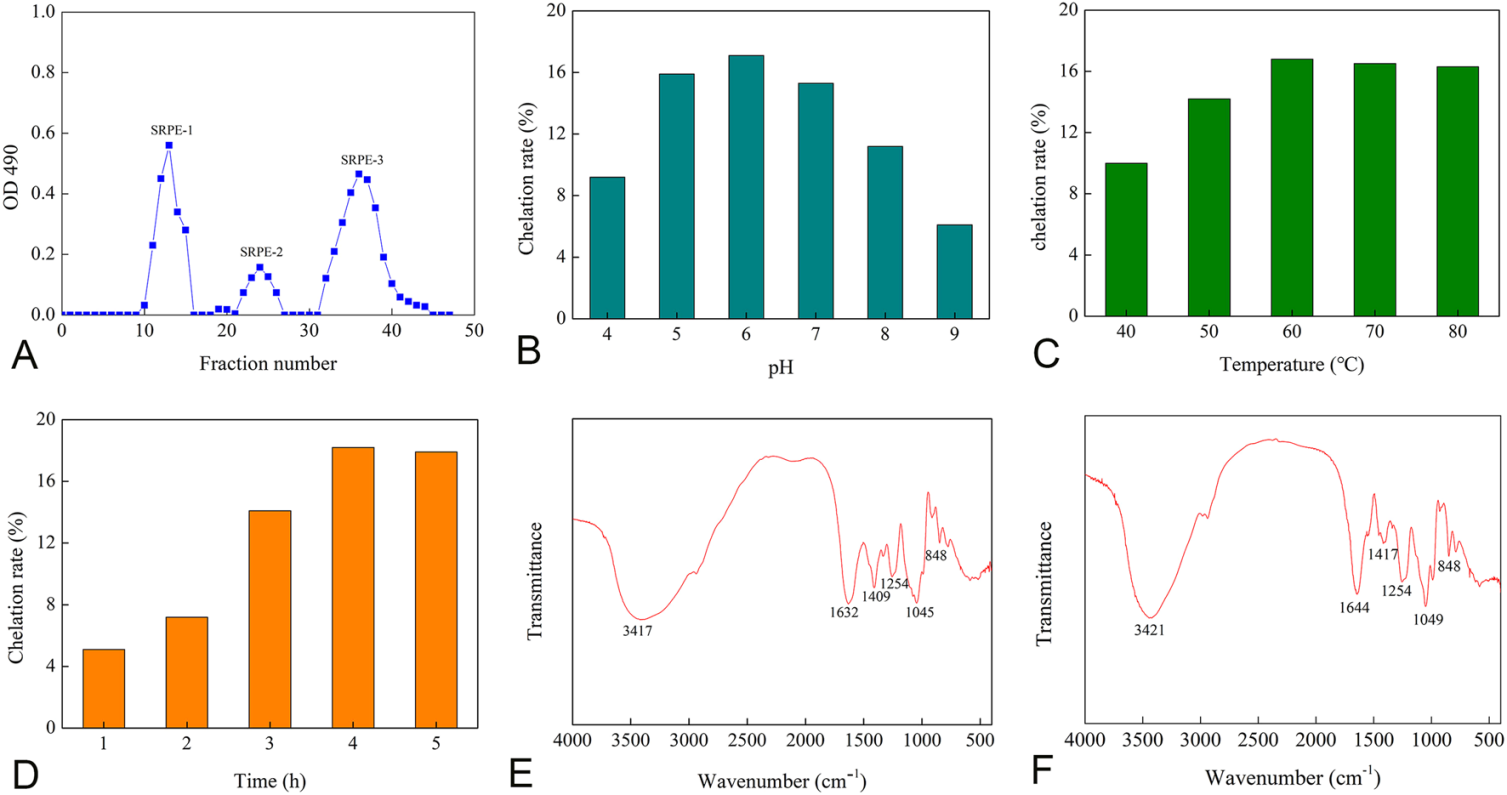
**A** Purification of enzymatic product of SRP. **B**–**D** The effect of pH (**B**), temperature (**C**), and time (**D**) on the Cr(III) chelation rate of SRPE-3. **E**, **F** Fourier transform infrared spectroscopy spectrum of SRPE-3 (**E**) and SRPE-3-Cr(III) complex (**F**). Chelation condition: **B-**50 °C, 3 h; **C**-pH 6, 3 h; **D**-pH 6, 60 °C

The enzymatic product of SRP was divided into three independent elution fractions after purification with Sephacryl S-300 HR column (Fig. 1A). According to the elution peak sequence obtained after concentration and dialysis, the three fractions were nominated as SRPE-1, SRPE-2, and SRPE-3. Based on the peak area in sephacryl S-300 HR chromatography, the mass ratio of SRPE-1, SRPE-2, and SRPE-3 was 1.5:0.6:2.8.

The SRPE-1 and SRPE-2 appeared as white powder, while the SRPE-3 was a little yellow. The sugar content of SRPE-3 was higher than that of SRPE-1 and SRPE-2 because of its low protein content (Table 1). Besides, the SRPE-3 with the lowest average molecular weight showed the highest content of sulfate ester. Analysis by High Performance Liquid Chromatography (HPLC) indicated that Rha was the main monosaccharide of the three fractions, while the molar ration percentage of Rha in SRPE-3 was higher than that of SRPE-1 and SRPE-2. According to Yu et al. (2017), sulfate ester is at the C-3 position of Rha. Consequently, the highest molar ratio percentage of Rha might contribute the highest content of sulfate ester of SRPE-3. In view of the best aqueous solubility of SRPE-3 and preliminary experiments examining adsorption of SRPE-1, SRPE-2, and SRPE-3, the SRPE-3 was selected to be used in the subsequent experiment.

**Table 1:** Characterization of SRPE-1, SRPE-2, and SRPE-3

	SRPE-1	SRPE-2	SRPE-3
Total sugar (%)	62.23	64.45	68.45
Sulfate ester (%)	20.34	21.12	27.04
Protein content (%)	4.12	3.01	1.81
Average molecular weight (kDa)	599.24	375.49	171.88
Rha (%)	48.56	49.89	55.20
GlcA (%)	17.42	18.58	15.25
Glu (%)	20.45	16.08	19.38
Xyl (%)	13.57	15.45	10.17

Rha, rhamnose; GlcA, galacturonic acid; Glu, glucose; Xyl, xylose

### Effects of factors on chelation of SRPE-3-Cr(III) complex

The reactions were conducted to evaluate the potential association between pH and Cr(III) chelation rate of SRPE-3 (Fig. 1B). Chelation rate increased from 9.1 to 17.3% when pH increased from 4.0 to 6.0. However, a further increase in pH resulted in a significant decrease in the rate of chelation. The Cr(III) chelation rate of SRPE-3 decreased to 6.1% when the reaction pH was 9.0. Due to the important role of temperature in chelation, the effect of temperature (40–80 °C) on the Cr(III) chelation rate of SRPE-3 was determined (Fig. 1C). The Cr(III) chelation rate of SRPE-3 increased gradually from 40 to 60 °C then decreased gradually from 60 to 80 °C. As shown in Fig. 1D, under optimal conditions (i.e., pH 6.0 and temperature 60 °C), a rapidly increasing chelation rate was observed as chelation time increased from 1 to 4 h, and then decreased slightly after 4 h. Considering these results, time 4 h was chosen to conduct our experiments.

### Comparison of SRPE-3 and SRPE-3-Cr(III) characteristics by FT-IR

The FT-IR spectra of SRPE-3 and SRPE-3-Cr(III) are presented in Fig. 1E, F. The stretch vibration band of O–H appeared at 3421/cm in SRPE-3 (Fig. 1E). However, in the SRPE-3-Cr(III) complex, the absorption band not only shifted to 3421/cm but also narrowed (Fig. 1F), indicating the formation of a coordination bond between Cr(III) and hydroxyl group (Cui et al. 2019). The characteristic absorption bands of SRPE-3 at 1632/cm and 1254/cm were generated by the stretching vibrations of C=O, and S=O, respectively (Yu et al. 2017). The C=O-stretching vibration shifted to 1644/cm after the chelation with Cr(III) (Fig. 1F), indicating the C=O group participated in the chelation reaction. However, the stretching vibration absorption band of S=O showed no shift, indicating the insignificant involvement of S=O in Cr(III) chelation of SRPE-3.

### SRPE-3-Cr(III) decreased the ratio of adipose tissue in the body

Adipose tissue plays an important role in systemic energy storage management as well as in many other processes (Wang et al. 2018). As depicted in Table 2, body mass, adipose mass, and body fat ratio in the DC group were remarkably greater than those in the NC group (*P* < 0.01), indicating that the HFSD used in this study significantly increased the accumulation of fat in mice. Treatment with SRPE-3-Cr(III) and chromium picolinate was observed to attenuate body fat ratio compared with no treatment, whereas treatment with SRPE-3 and chromium trichloride hexahydrate showed no obvious attenuation.

**Table 2:** Effect of SRPE-3-Cr(III) on body mass and adipose mass in T2DM mice (*n* = 9)

Groups	Body mass (g)	White adipose mass (g)	Brown adipose mass (g)	Epididymal adipose mass (g)	Perirenal adipose mass (g)	Ratio of adipose in body (%)
Normal control (NC)	25.77 ± 1.08	0.34 ± 0.16	0.06 ± 0.01	0.24 ± 0.06	0.04 ± 0.02	2.48 ± 0.18
Diabetic (DC)	29.56 ± 1.64^##^	0.86 ± 0.22^##^	0.19 ± 0.09^##^	0.59 ± 0.24^##^	0.21 ± 0.07^##^	6.26 ± 1.07^##^
Positive control (PC)	26.99 ± 2.11**	0.32 ± 0.18**	0.05 ± 0.01**	0.34 ± 0.08**	0.07 ± 0.02**	2.89 ± 0.39**
Negative control (CT)	28.66 ± 1.68	0.82 ± 0.17	0.15 ± 0.08	0.52 ± 0.09	0.16 ± 0.07*	5.76 ± 0.31
Low-SRPE-3	28.96 ± 2.19	0.85 ± 0.20	0.17 ± 0.07	0.54 ± 0.12	0.18 ± 0.06	6.07 ± 0.91
High-SRPE-3	28.84 ± 1.35	0.87 ± 0.15	0.18 ± 0.08	0.57 ± 0.08	0.17 ± 0.08*	6.21 ± 0.27
Low-SRPE-3-Cr(III)	26.59 ± 0.68**	0.47 ± 0.19**	0.05 ± 0.01**	0.37 ± 0.09**	0.06 ± 0.04**	3.57 ± 0.50**
High-SRPE-3-Cr(III)	26.83 ± 1.30**	0.40 ± 0.15**	0.06 ± 0.01**	0.34 ± 0.09**	0.08 ± 0.03**	3.28 ± 0.46**

^##^*P* < 0.01 versus the control group. **P < 0.01 versus the model group. *P < 0.05 versus the model group

### Effect of SRPE-3-Cr(III) on blood glucose levels

Table 3 shows the blood glucose level of the T2DM mice induced by consumption of an HFSD. The concentration of glycosylated hemoglobin (HbA1c) is a long-term indicator of diabetic control (Krzysik et al. 2011). An HFSD induced elevations in fasting blood glucose (FBG). HbA1c and glycosylated serum protein levels significantly decreased with SRPE-3-Cr(III) treatment. The blood glucose level was significantly reduced with the administration of SRPE-3-Cr(III) (10 and 30 mg/kg) compared to the DC group, while SRPE-3 treatment groups did not change significantly. Chromium picolinate treatment (*P* < 0.01) played a part in improvement of the damaged blood level of T2DM mice. These results indicate that SRPE-3-Cr(III) exhibits a significant antihyperglycemic effect.

**Table 3:** Effect of SRPE-3-Cr(III) on blood glucose levels in T2DM mice (*n* = 9)

Groups	FBG (mmol/L)	HbA1c (mmol/L)	Glycosylated serum protein (mmol/L)
Normal control (NC)	6.01 ± 0.41	18.34 ± 1.16	1.97 ± 0.25
Diabetic (DC)	12.56 ± 0.64^##^	27.86 ± 1.22^##^	3.18 ± 0.24^##^
Positive control (PC)	7.09 ± 1.11**	20.32 ± 1.18**	2.38 ± 0.11**
Negative control (CT)	11.66 ± 0.68	28.82 ± 1.17	3.13 ± 0.28
Low-SRPE-3	11.03 ± 1.19	27.85 ± 1.20	3.18 ± 0.17
High-SRPE-3	12.04 ± 1.35	26.87 ± 1.15	3.05 ± 0.19
Low-SRPE-3-Cr(III)	7.59 ± 0.88**	21.47 ± 1.19**	2.54 ± 0.12**
High-SRPE-3-Cr(III)	7.83 ± 1.30**	20.84 ± 1.15**	2.11 ± 0.15**

^##^*P* < 0.01 versus the control group. **P < 0.01 versus the model group

### Effect of SRPE-3-Cr(III) on serum TC, TG, LDL-C, and HDL-C levels in T2DM mice

**Fig. 2:**
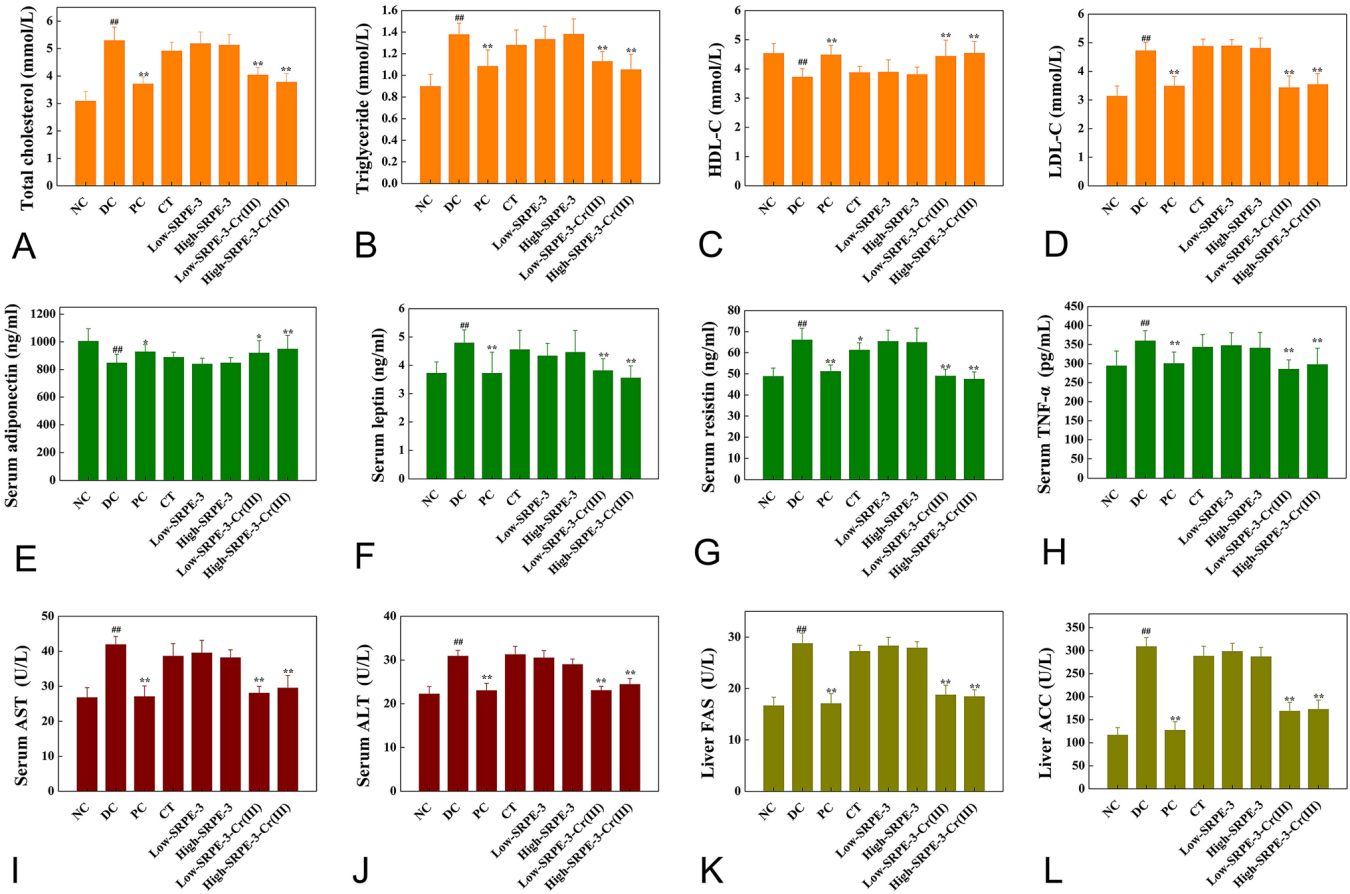
Effect of SRPE-3-Cr(III) on concentration of serum TC (**A**), TG (**B**), LDL-C (**C**), HDL-C (**D**), adiponectin levels (**E**), leptin (**F**), resistin (**G**), TNF-α (**H**), activity of alanine aminotransferase (ALT) (**I**), activity of aspartate aminotransferase (AST) (**J**), activity of fatty acid synthase (FAS) (**K**), and activity of acetyl-CoA carboxylase (ACC) (**L**) in T2DM mice (*n* = 9). ^##^*P* < 0.01 relative to the control group. **P < 0.01 relative to the model group. *P < 0.05 relative to the model group

The increase of TC, TG, and LDL-C levels and the decrease of HDL-C levels lead to hyperlipidemia. Figure 2A–D shows the effect of different treatments on TC, TG, LDL-C, and HDL-C levels in T2DM mice. Our results revealed that the levels of serum TC, TG, and LDL-C (*P* < 0.01) in the DC group were significantly higher than that of mice in the NC group. However, mice in the DC group showed a significant decrease in the level of serum HDL-C (*P* < 0.01) compared to the NC group. Both SRPE-3-Cr(III) and chromium picolinate administration showed a significant decrease in the levels of serum TC, TG, and LDL-C and a significant increase in the level of HDL-C after an 11-week treatment when compared with the diabetic group (*P* < 0.01). This suggested that SRPE-3-Cr(III) had a beneficial effect on decreasing the blood lipid level.

### SRPE-3-Cr(III) normalized adipokine levels

Adipokines have been shown to play an important role in the physiology and pathophysiology of insulin sensitivity in T2DM (Hu et al. 2014a, b). As presented in Fig. 2E–H, the serum levels of leptin, resistin, and TNF-α of the diabetic mice were higher than those of the NC group (*P* < 0.01). SRPE-3-Cr(III) administration (10 and 30 mg/kg) notably reduced the serum levels of leptin, resistin, and TNF-α, and increased adiponectin (*P* < 0.01) compared to the values in the DC group. Furthermore, the serum adipokine levels of the SRPE-3-Cr(III) group were similar to those of the positive control group. This indicates that under our experimental conditions, SRPE-3-Cr(III) can normalize adipokines production.

### Determination of serum AST and ALT activities

Enhancement of liver enzyme activities in the serum are usually defined as a marker of liver damage (Shituleni et al. 2016). As shown in Fig. 2I, J, AST and ALT activities significantly increased with HFSD (*P* < 0.01). In contrast, there was a significant decrease in AST and ALT activities after 10 and 30 mg/kg SRPE-3-Cr(III) treatments (*P* < 0.01). Improvements of AST and ALT activities of T2DM mice were also observed after chromium picolinate treatment (*P* < 0.01).

### Effect of SRPE-3-Cr(III) on hepatic lipid metabolism-related enzyme activity

The activities of hepatic lipid metabolism-related enzymes are shown in Fig. 2K, L. The FAS and ACC activities were higher in the untreated hyperlipidemic than in the normal control mice (*P* < 0.01). Treatment with SRPE-3-Cr(III) (10 and 30 mg/kg) and chromium picolinate were shown to significantly suppress the activities of FAS and ACC in the hyperlipidemic mice (*P* < 0.01).

### Microscopic structures of pancreas islets

**Fig. 3:**
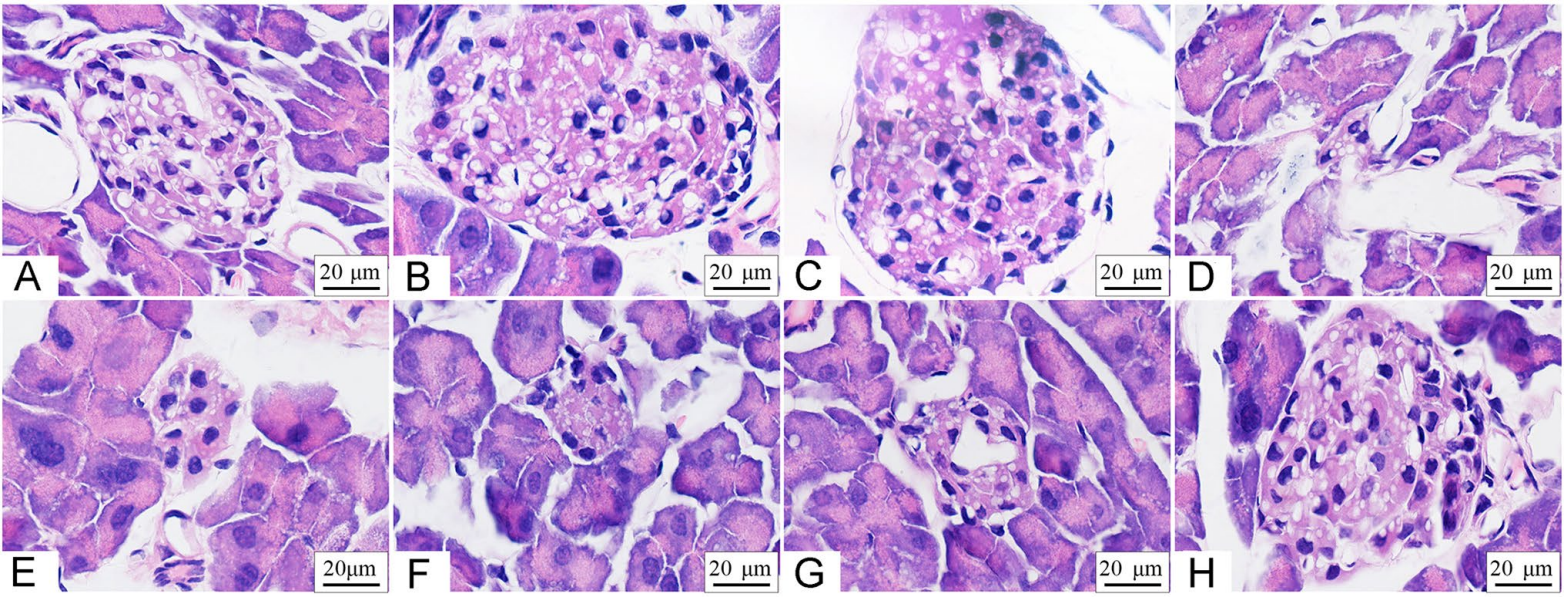
Microstructure of pancreatic islets in T2DM mice (HE stain, 100×). **A** Normal control; **B** positive control; **C** low-SRPE-3; **D** low-SRPE-3-Cr(III); **E** diabetic; **F** negative control; **G** high-SRPE-3; **H** high-SRPE-3-Cr(III)

Pancreatic β-cells are specialized cells that secrete insulin to balance blood glucose (Hu et al. 2014a, b). In the DC group, various pathological changes including atrophy of pancreas islet, necrocytosis, and inhomogeneous distribution of nuclear chromatin were observed (Fig. 3). Compared with the DC group, there was no significant change in the appearance of pancreatic islets in neither the low SRPE-3 group nor the high SRPE-3 group. To the contrary, after treatment with SRPE-3-Cr(III), histological changes in insulin resistant mice were significantly reduced and the islets returned to a normal rounded appearance.

## Discussion

Chromium(III) is required for optimal insulin activity and normal carbohydrate and lipid metabolism (Sahin et al. 2007). It affects glucose metabolism by enhancing its involvement in the mechanism of insulin signal amplification (Sharma et al. 2011). Chromium has the potential to regulate the Glucose Transporters 4 (GLUT4) glucose transporter and enhance activity of the cellular energy sensor 5’ AMP-activated protein kinase (Hoffmana et al. 2014). Chromium can also reduce plasma membrane cholesterol by lowering the biological effect of tyrosine phosphatase 1B and irritating the transfer of the GLUT4 glucose transporter (Wang et al. 2006). While the role of chromium(III) in improving lipid level and insulin action remains to be further studied. In addition, chromium may also regulate lipid metabolism by reducing the expression of sterol regulatory element-binding protein (SERBP-1) (Chen et al. 2006). However, the bioavailability and bioaccumulation of metal elements in organic form are better than those in inorganic form (Tan et al. 2001). In light of the fact that polysaccharides are a good organic ligand for metal elements (Chi et al. 2021), it is necessary to determine the optimal chelating conditions of SRPE-3 and Cr(III). Li et al. (2019), reported that an alkaline reaction system led to a slow decrease in the chelation rate of *Ganoderma lucidum* polysaccharide, which was different from this result. This difference may be due to the different substituent groups in *Ganoderma lucidum* polysaccharide, in which there were only carboxylic and hydroxyl group. SRPE-3 molecules move too fast in a reaction system at high temperature, reducing the chance of binding with Cr(III) and leading to a low chelation rate (Guo et al. 2019).

Except for hyperglycemia and insulin resistance, the evolution of T2DM is frequently combined with dyslipidemia, often an important determinant of the course and status of the diabetic patients (Huang et al. 2015). In our study, increase in serum TC, TG, LDL-C levels, and decrease in HDL-C level in T2DM mice were observed, suggesting that an HFSD could induce oxidative stress. Examination of ABCA1 trafficking revealed that the presence of chromium could prevent the decrease of plasma membrane ABCA1 by hyperinsulinemia conditions relevant to onset of disease (Sealls et al. 2011). On the other hand, chromium could improve insulin sensitivity and, probably, contribute to the reduction in triglyceride hydrolysis in adipocytes (Krzysik et al. 2011). In animal studies, some polysaccharides–chromium complexes, such as *Grifola frondosa* polysaccharide-chromium(III) complex and *Momordica charantia* polysaccharide–chromium(III) complex, could inhibit low-density lipoprotein oxidation and have an overall positive effect on lipid metabolism and cholesterol (Guo et al. 2019; Zhang et al. 2019). Our study showed that administration of SRPE-3-Cr(III) resulted in a decrease in serum TC, TG and LDL-C, suggesting that SRPE-3-Cr(III) could attenuate the disorder of lipid metabolism.

Adipose tissue is the main tissue supplying excess nutrient storage for triacylglycerols and also generating various secreted proteins called adipokines (Kwon and Pessin 2013). These proteins regulate diverse metabolic functions in an autocrine, paracrine, or endocrine fashion. In fact, adipokines possibly act to change insulin sensitivity of insulin-targeted organs (Gan et al. 2012). Although leptin, TNF-α, resistin, and adiponectin are all representative adipokines, the first three are positively correlated with insulin resistance and adiponectin is negatively correlated (Bokarewa et al. 2005). In this study, serum resistin, TNF-α, and leptin levels were dramatically reduced by SRPE-3-Cr(III) treatment, indicating SRPE-3-Cr(III) alleviated insulin resistance by inhibiting the secretion of serum resistin, leptin, and TNF-α from adipocytes. The upregulation of adiponectin in T2DM mice and the ability of chromium to reconcile the elevated levels of adiponectin observed in this study support the hypothesis that SRPE-3-Cr(III) could down-regulate inflammation associated with diabetes. The microstructure of islet also showed that SRPE-3-Cr(III) could repair β-islet cells, inhibit the apoptosis of β-islet cells, and restore the normal morphology of the cells.

FAS and ACC are known to be the two important enzymes that regulate the occurrence of lipogenesis. After consumption of a high-carbohydrate diet, FAS and ACC are activated to promote glycolysis, leading to increased levels of endogenous fatty acids (Hu et al. 2017). Sadeghi et al. (2015) have previously reported that supplementation of domestic goat kids (Capra hircus) with 1.5 mg/day chromium decreased the accumulation of fat in adipose tissues, such as the liver, subcutaneous fat, and visceral fat, through down-regulation of the gene encoding ACC1 enzyme. Wang et al. (2014) also reported that supplemental chromium from chromium-loaded chitosan nanoparticles positively affected lipid catabolism in finishing pigs through decreasing the activity of FAS in adipose tissues. These results are in accordance with the outcomes of this study. The decrease of FAS and ACC in the liver of T2DM mice indicated that SRPE-3-Cr(III) had an effect on stimulating the degradation of fatty acid, while weakening the fatty acid synthesis.

## Materials and methods

### Materials and chemicals

*Enteromorpha prolifera* was collected from the coasts of Qingdao, China. Picolinic acid chromium(III) salt was purchased from Energy Chemical (Shanghai, China). All other chemicals and reagents were of analytical grade.

### Preparation and purification of enzymatic product of SRP

The SRP was extracted according to a method described by Yu et al. (2017). The SRP was degraded by purified DPE-L according to Li et al. (2016) with some modifications. Briefly, 100 ml SRP with a concentration of 15 mg/ml was incubated with 50 U purified DPE-L for 6 h at 35 °C. The reaction was stopped using a boiling water bath for 5 min. Next, the SRP hydrolysate was centrifuged at 10,000 *g* for 15 min. The supernatant was precipitated by adding fivefold-volume 95% ethanol. The precipitate was collected and dissolved with water. After centrifugation, desalting, and lyophilization, the enzymatic product of SRP was prepared then injected into Sephacryl S-300 HR column (1.0 cm× 100 cm). Samples were then eluted using 50 mmol/L NaCl with flow rate of 0.2 ml/min. The collected fraction was desalted with the Sephadex G-10 column (2.0 cm× 60 cm) and eluted with ultrapure water.

### General analysis of SRPE-1, SRPE-2, and SRPE-3

The content of protein, total sugar, sulfate ester, and uronic acid were determined by the Bradford assay (Bradford et al. 1976), phenol–sulphuric acid method (Dubois et al. 1956), BaCl_2_-gelatin method (Kawai et al. 1969), and 3-phenylphenol method (Blumenkrantz and Asboe-Hansen 1973), respectively. The molecular weight of SRPE-1, SRPE-2, and SRPE-3 was measured using gel permeation chromatography (GPC) with PL Aquagel-OH 50 columns (30 cm × 7.8 mm, Aligent, USA) on Agilent 1260 HPLC system and eluted with 0.2 mol/L NaNO_3_ containing 0.05 mol/L NaH_2_PO_4_ at 0.6 ml/min flow rate. Dextrans with different molecular weights (1185, 670, 270, 15,050, and 10 kDa) were used to determine the molecular weight of fractions. The molecular weight and monosaccharide of three fractions were determined according to the method described by Yu et al. (2017).

### Optimization of the synthesis conditions for SRPE-3-Cr(III)

The effect of conditions including pH (4–9), temperature (40–80 °C), and time (1–5 h) on the synthesis of SRPE-3 chromium(III) (SRPE-3-Cr(III)) was then determined. Briefly, 0.5 mol/L CrCl_3_ was added dropwise under continuous stirring to 10 mg/ml SRPE-3 solution. During the process, 1 mol/L NaOH or HCl was added to control pH of the system to different pHs. Next, the solution was heated to different temperatures for different time, respectively. The supernatant was obtained after centrifugation at 4000 r/min for 15 min and dialyzed using dialysis tubing (3500 Da MWCO membrane) in distilled water for 36 h to remove the free Cr(III). Finally, the dialysate was freeze-dried to produce SRPE-3-Cr(III).

### Characterization of SRPE-3-Cr(III)

#### Quantification of chromium in SRPE-3-Cr(III)

The content of chromium in SRPE-3-Cr(III) complex was determined by inductively coupled plasma mass spectrometry (ICP-MS) as described by Huang et al. (2005).

The chelation rate of SRPE-3 was calculated according to the following equation:

$$ {\text{Chelationrate}}\, \left( {\text{\%}}\right) = \frac{C}{m} \times 100{\text{\%,}} $$

where *C* is the total content of chromium (mg) in SRPE-3-Cr(III) complex and *m* is the total mass of SRPE-3 (mg).

#### Fourier transform infrared (FT-IR) spectroscopy

The FI-IR absorption of SRPE-3 and SRPE-3-Cr(III) was recorded using an FT-IR spectrometer (Thermofisher 6700, USA) in the region of 4000–400/cm according to the research of Zhao et al. (2021).

### Animals and experimental design

Male C57BL/6 J mice, 4–5 weeks, 18–20 g, were purchased from Jinan Pengyue Experimental Animal Breeding Co., Ltd. (Shandong, China, License ID: SCXK2014-0007). They were housed at 23 ± 1 °C and humidity of 44.5–51.8% with a 12:12 h light–dark schedule in Ocean University of China. All experimental protocols were approved by the Committee on Experimental Animal Nursing Ethics of Ocean University of China (certificate no. SYXK20120014). All animal experiments were carried out in accordance with internationally validated guidelines of the Standards for Laboratory Animals of China (GB 14922-94, GB 14923-94, and GB/T 14925-94).

Animals were randomly assigned to 8 groups of 9 animals each. As described in Table 4, the normal control group (NC) was maintained on a low-fat, low-sucrose diet (LFSD), while the T2DM-model mice were established by being fed an HFSD (Hu et al. 2014a, b). Both diets were not supplemented with chromium. Blood samples were collected from the tail vein to determine fasting blood glucose (FBG). Mice with FBG level greater than 11.1 mmol/L were considered diabetic mice so used in this study (Zhang et al. 2010).

**Table 4:** Composition of animals’ experimental diets

Ingredients	LFSD (g/kg)	HFSD (g/kg)
Casein	200	200
Cornstarch	690	290
Corn oil	50	50
Mineral mix	40	40
Vitamin mix	10	10
Cellulose	5	5
Choline bitartrate	3	3
DL-methionine	2	2
Sucrose	/	200
Lard	/	200
Chromium	/	/

The mineral mix and vitamin mix were prepared according to the AIN-93 recipe

After establishment of the T2DM model, mice were randomly assigned to normal control, model control, positive control, negative control, low SRPE-3, high SRPE-3, low SRPE-3-Cr(III), and high SRPE-3-Cr(III) groups. The specific doses of the samples are shown in Table 5. Testing samples were dissolved in distilled deionized water and administered mice by orogastric cannula for 77 days. All groups were fed with an HFSD except the normal control group, which was fed a normal diet. Body weight was measured weekly.

**Table 5:** Grouping design and dosage of type 2 diabetic experiment

Groups	Samples	Dose (mg/kg b.w.)	Equivalent to Cr^3+^ (µg/kg b.w.)
Normal control (NC)	NaCl (0.9%)		
Diabetic (DC)	NaCl (0.9%)		
Positive control (PC)	Chromium(III) picolinate	6.8	840
Negative control (CT)	CrCl_3_·6H_2_O	4.3	840
Low-SRPE-3	SRPE-3	10	
High- SRPE-3	SRPE-3	30	
Low-SRPE-3-Cr(III)	SRPE-3-Cr(III)	10	280
High-SRPE-3-Cr(III)	SRPE-3-Cr(III)	30	840

### Determination of biochemical parameters

At the end of the experimental period, all mice were under fasting conditions for 12 h and allowed to drink water freely. Blood was obtained from the orbital sinus. Mice were sacrificed by cervical spine without the use of anesthesia. The blood samples were centrifuged immediately at the condition of 3000 *g*, 10 min, 4 °C to obtain the serum, which was used to determine adipokines levels using ELISA kits (Invitrogen, Carlsbad, CA, USA). The serum TC, TG, LDL-C, HDL-C, ALT, and AST were estimated using a reagent kit according to the instructions of the manufacturer (Jiancheng Bioengineering Institute, Nanjing, China). Glycosylated hemoglobin (HbA1c) levels and glycosylated serum protein levels were determined using commercial kits (Biosino, Beijing, China).

### Measurement of hepatic lipid metabolism-related enzyme activity

The liver sample (100 mg) was homogenized in ice-cold 50 mmol/L PBS solution (1:9, w/v); the supernatant was obtained by centrifugation at 5000 *g* for 5 min at 4 °C. Activities of fatty acid synthase (FAS) and acetyl-CoA carboxylase (ACC) were estimated using ELISA kits (Invitrogen, Carlsbad, CA, USA) according to the manufacturer’s instructions. The protein content was assayed by a total protein quantitative assay kit (Jiancheng Bioengineering Institute, Nanjing, China).

### Histopathological examination

The pancreatic tail was fixed in 4% paraformaldehyde, paraffin embedded, sliced, and stained with hematoxylin and eosin (HE). The microstructures of islets were observed and photographed by optical microscopy (BH-2 Olympus, Japan).

### Statistical analysis

Data were expressed as a mean ± standard deviation. Statistical analyses were performed by one-way analysis of variance (ANOVA) followed by the least significant difference (LSD) test. *P* value of less than 0.05 was considered statistically significant.

## Data Availability

All data generated or analysed during this study are included in this published article (and its supplementary information files).

## Abbreviations

HDL-C: High-density lipoprotein cholesterol
TG: Triglyceride
TC: Total cholesterol
LDL-C: Low-density lipoprotein cholesterol
HbA1c: Glycosylated hemoglobin
AST: Aspartate aminotransferase
ALT: Alanine aminotransferase
FAS: Fatty acid synthase
ACC: Acetyl-CoA carboxylase
